# Probabilistic atlases of default mode, executive control and salience network white matter tracts: an fMRI-guided diffusion tensor imaging and tractography study

**DOI:** 10.3389/fnhum.2015.00585

**Published:** 2015-11-03

**Authors:** Teresa D. Figley, Navdeep Bhullar, Susan M. Courtney, Chase R. Figley

**Affiliations:** ^1^Department of Radiology, University of ManitobaWinnipeg, MB, Canada; ^2^Division of Diagnostic Imaging, Health Sciences CentreWinnipeg, MB, Canada; ^3^Neuroscience Research Program, Kleysen Institute for Advanced MedicineWinnipeg, MB, Canada; ^4^Department of Psychological and Brain Sciences, Johns Hopkins UniversityBaltimore, MD, USA; ^5^Solomon H. Snyder Department of Neuroscience, Johns Hopkins UniversityBaltimore, MD, USA; ^6^F. M. Kirby Research Center for Functional Brain Imaging, Kennedy Krieger InstituteBaltimore, MD, USA; ^7^Biomedical Engineering Graduate Program, University of ManitobaWinnipeg, MB, Canada

**Keywords:** brain atlas, connectivity, connectome, default mode network, executive control network, salience network, white matter

## Abstract

Diffusion tensor imaging (DTI) is a powerful MRI technique that can be used to estimate both the microstructural integrity and the trajectories of white matter pathways throughout the central nervous system. This fiber tracking (aka, “tractography”) approach is often carried out using anatomically-defined seed points to identify white matter tracts that pass through one or more structures, but can also be performed using functionally-defined regions of interest (ROIs) that have been determined using functional MRI (fMRI) or other methods. In this study, we performed fMRI-guided DTI tractography between all of the previously defined nodes within each of six common resting-state brain networks, including the: dorsal Default Mode Network (dDMN), ventral Default Mode Network (vDMN), left Executive Control Network (lECN), right Executive Control Network (rECN), anterior Salience Network (aSN), and posterior Salience Network (pSN). By normalizing the data from 32 healthy control subjects to a standard template—using high-dimensional, non-linear warping methods—we were able to create probabilistic white matter atlases for each tract in stereotaxic coordinates. By investigating all 198 ROI-to-ROI combinations within the aforementioned resting-state networks (for a total of 6336 independent DTI tractography analyses), the resulting probabilistic atlases represent a comprehensive cohort of functionally-defined white matter regions that can be used in future brain imaging studies to: (1) ascribe DTI or other white matter changes to particular functional brain networks, and (2) compliment resting state fMRI or other functional connectivity analyses.

## Introduction

Stereotaxic brain atlases play an important role in neuroscience and neuroimaging research. Warping (or “normalizing”) images to a standardized brain template provides an effective and principled way to report anatomical regions of interest (ROIs), perform quantitative analyses, and directly compare data acquired from different subjects and/or patient populations. The first widely-adopted template was based on the brain of a single subject (Talairach and Tournoux, 1988). However, shortly thereafter a group of researchers from Canada, The United States, and Germany formed the International Consortium for Brain Mapping (ICBM), which set out to create standardized human brain atlases that were based on high-resolution anatomical MRI data from large populations of healthy control subjects (Evans et al., 1992, 1993; Collins et al., 1994; Mazziotta et al., 1995). These templates have since been adopted by neuroimaging researchers around the world for normalizing individual data for group analyses, and to this day are distributed with many popular image processing and fMRI analysis software packages (c.f., Brett et al., 2002; Lancaster et al., 2007). However, although these anatomical atlases serve as convenient and effective templates for linear normalization and cross-subject cortical alignment, they provide somewhat limited information about subcortical structures in general, and white matter in particular (Toga et al., 2006). For this reason, focus has also been placed on generating stereotaxic atlases that include anatomically-segmented cortical and subcortical structures (Shattuck et al., 2008), as well as those that are specific to white matter anatomy [e.g., the Johns Hopkins “*Adam”* (Wakana et al., 2004) and “*Eve”* atlases (Mori et al., 2008; Oishi et al., 2008, 2009), in which the cerebral white matter has been parcellated into more than 175 distinct anatomical regions]. Moreover, by examining white matter connectivity between various anatomically-defined seed regions, diffusion tensor imaging (DTI) and fiber tracking (or “tractography”) methods have been used to generate both probabilistic (Hua et al., 2008; Zhang et al., 2010) and non-probabilistic (Catani and Thiebaut de Schotten, 2008; Catani et al., 2012) white matter atlases.

In parallel to these advances, the burgeoning fields of resting state fMRI (rs-fMRI) and functional connectivity analysis have exploded in popularity—leading to the identification of intrinsic correlations between distributed cortical regions that appear to form functionally-connected brain networks [see Fox and Raichle, 2007 and Smith et al., 2013 for detailed reviews]. The earliest rs-fMRI reports astutely observed that low frequency (<0.1 Hz) correlations between cortical regions were likely manifestations of intrinsic connections that could be used to identify functional brain networks (Biswal et al., 1995). Based on this premise, a large (and growing) number of resting state networks have been identified, including: (1) task-negative networks such as the so-called default mode network (DMN) (Greicius et al., 2003; Fox et al., 2005; Buckner et al., 2008), which are consistently suppressed during many cognitive and perceptual tasks, and (2) networks that show positive activation during these same tasks, such as the executive control network (ECN) and the salience network (SN) (Seeley et al., 2007). Owing to these and other advances, the prevailing views in systems and cognitive neuroscience have undergone somewhat of a paradigm shift (Friston, 2002). Where it was previously assumed that neural processing for different tasks was carried out in isolated brain regions, the preponderance of evidence now supports the view that sensory, motor and cognitive processing all rely on distributed, large-scale brain networks (Bressler and Menon, 2010).

Based on this network model, it stands to reason that specific brain functions (e.g., cognitive processes) depend on the structural and functional integrity of both the cortical regions comprising the “nodes” of each network, and the white matter pathways connecting these nodes (Sporns et al., 2005). A number of studies have therefore sought to directly examine the relationships between structural and functional connectivity within the brain networks of healthy control subjects (Greicius et al., 2009; Honey et al., 2009, 2010; Hermundstad et al., 2013)—which have shown that white matter structural properties, such as the number of white matter streamlines between regions, are indicative of resting-state and task-based functional correlations (see Wang et al., 2015 for a recent and comprehensive review on structure-function relationships)—while others have speculated about the associations between white matter integrity and functional connectivity changes in patient populations (Damoiseaux and Greicius, 2009; Hawellek et al., 2011; Uddin, 2013). However, while the cortical nodes of these networks can be readily identified and delineated using fMRI (as evidenced by their relatively consistent positions across individuals and studies) and their locations and extents have been previously reported in stereotaxic coordinates (Shirer et al., 2012), the corresponding white matter regions “belonging” to each network have not yet been defined.

This disparity—in our ability to localize cortical regions, but not the underlying white matter structures associated with these functional brain networks—imposes several limitations on the interpretation of DTI and other quantitative white matter imaging data. In particular, it makes direct comparisons between structural and functional connectivity extremely difficult, and completely prevents group-wise (e.g., patients vs. healthy controls) or regression (e.g., with age, gender, cognitive performance, or any other independent variable) analyses from ascribing region-of-interest (ROI) or voxel-wise white matter changes to a particular brain network or group of networks (i.e., similar to what is commonly done in contemporary fMRI studies).

To address these fundamental issues with the analysis and interpretation of diffusion and other quantitative white matter imaging data, the goals of the current study were to perform fMRI-guided DTI tractography on data acquired from a group of healthy adults to: (1) identify the specific white matter regions that are most likely to contain tracts between the nodes of six previously established and functionally-connected cortical networks—specifically the dorsal and ventral default mode networks (dDMN and vDMN), the left and right executive control networks (lECN and rECN), as well as the anterior and posterior salience networks (aSN and pSN)—and; (2) generate probabilistic white matter atlases based on these findings.

## Materials and methods

### Study participants

In order to achieve a sample size that was consistent with previous DTI-based (albeit, anatomically-defined) probabilistic white matter atlases (Hua et al., 2008; Oishi et al., 2009), 32 healthy volunteers (16 female) were recruited from the Baltimore community. Verbal screening was conducted to ensure that subjects had no history of neurological injury/disease, psychiatric illness, or substance abuse (including alcohol or tobacco). Of the 32 subjects, 19 were Caucasian, 9 were Asian, 2 were African American, and 2 were Hispanic. Subject age (29.9 ± 10.7 years), height (170.4 ± 8.3 cm), and weight (72.5 ± 16.2 kg) spanned a relatively broad range. In accordance with our study protocol, which was approved by the Institutional Review Boards of Johns Hopkins University and the Johns Hopkins Medical Institutions, all subjects provided written informed consent prior to study enrollment and were financially compensated for their participation.

### Data acquisition

All MRI data were acquired using a whole-body 3T Philips Achieva system and a 32-channel SENSE head coil *(Philips Healthcare, Best, The Netherlands)*. High-resolution T_1_-weighted images were acquired using a 3D MP-RAGE pulse sequence with the following parameters: TR = 7.93 ms; TE = 3.66 ms; Flip Angle = 8.00°; SENSE Factor (AP/RL/FH) = 2.4 (2.0/1.0/1.2); FOV (AP × FH × RL) = 212 × 150 × 172 mm; Spatial Resolution = 1.00 × 1.00 × 1.00 mm; Scan Duration = 4 min and 26 s. Purely T_2_-weighted (TR = 4162 ms; TE = 80 ms; Flip Angle = 90°; SENSE Factor = 2; FOV = 212 × 154 × 212 mm; Spatial Resolution = 1.10 × 1.10 × 2.20 mm), as well as fast T_2_-weighted Fluid Attenuated Inversion Recovery (T_2_-FLAIR) images (TR = 11000 ms; TI = 2800 ms; TE = 120 ms; Refocusing Angle = 120°; SENSE Factor = 1.75; FOV = 230 × 149 × 184 mm; Spatial Resolution = 1.00 × 1.20 × 5.00 mm) were also acquired and assessed by a board-certified radiologist to rule out structural abnormalities or other incidental findings.

Diffusion-weighted images were then acquired with a previously reported spin-echo echo-planar imaging (SE-EPI) pulse sequence (Farrell et al., 2007; Landman et al., 2007; Wakana et al., 2007) and the following parameters: 30 diffusion-weighted images (*b* = 700 s/mm^2^) with optimally oriented diffusion-encoding gradients (Jones et al., 1999; Skare et al., 2000); five reference images (*b* = 0 s/mm^2^); TR = 6904 ms; TE = 69 ms; Flip Angle = 90°; SENSE Factor = 2.5; FOV = 212 × 212 mm; Matrix Dimensions = 96 × 96 (zero-padded to 256 × 256); Number of Transverse Slices = 70 (no inter-slice gap); Slice Thickness = 2.2 mm; Scan Duration = 4 min and 16 s. Although pulse sequences with additional diffusion-encoding directions and higher *b*-values are able to use more sophisticated data reconstruction approaches – and therefore more reliably resolve complex fiber architectures (see *Study Limitations* for a more detailed explanation) – the acquisition parameters employed here are consistent with several previously published DTI-based white matter atlases (e.g., Wakana et al., 2004; Hua et al., 2008; Oishi et al., 2008, 2009; Zhang et al., 2010).

### Data analysis

**Figure 1:**
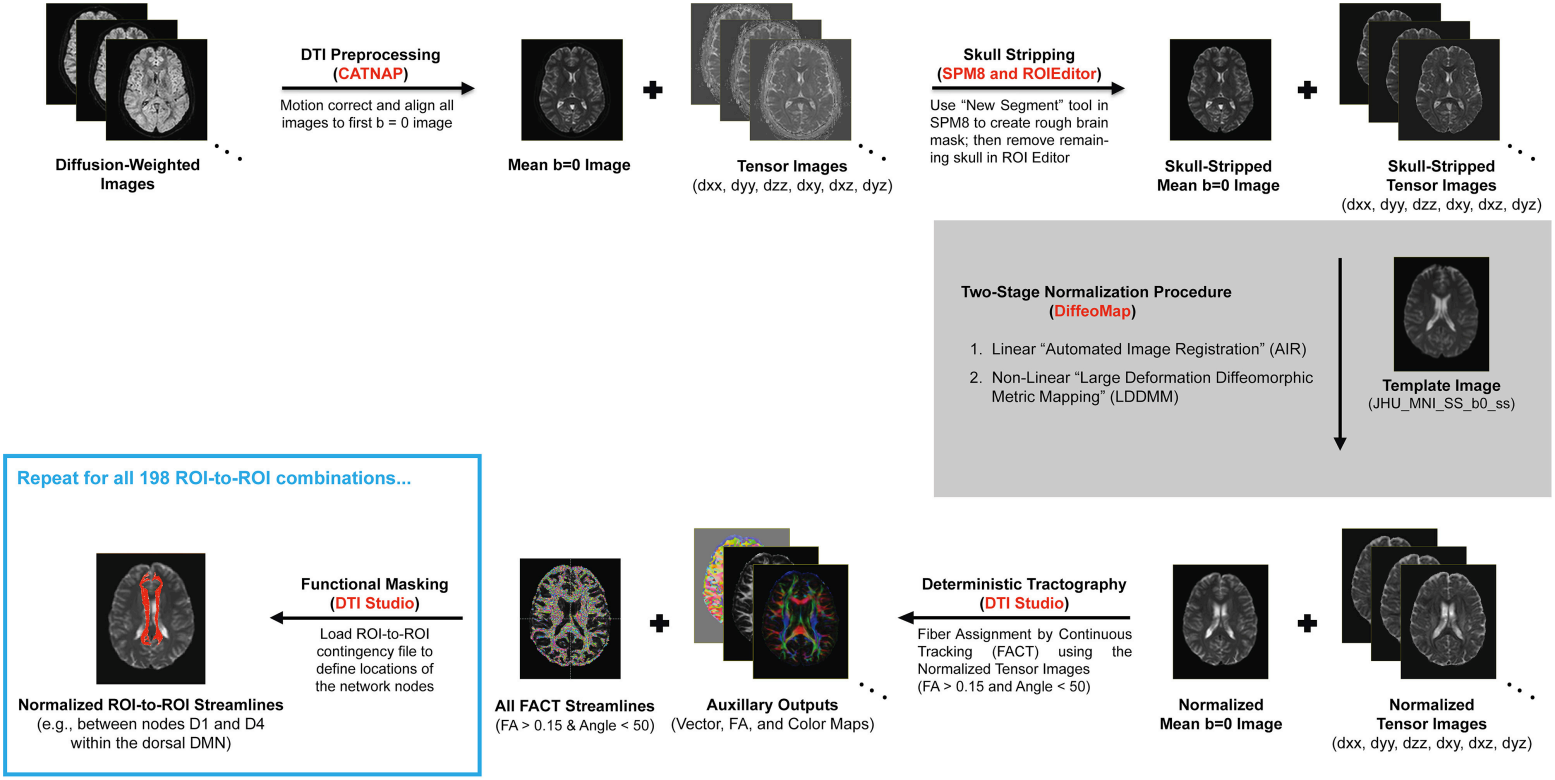
**Illustration of our DTI processing pipeline for each subject, which included multiple steps (black arrows) and made use of several different software packages (shown in red)**. After motion correcting and realigning the raw data, the mean *b* = 0 (s/mm^2^) and six tensor images were calculated, skull-stripped, and normalized to a standard anatomical template (i.e., the “JHU_MNI_SS_b0_ss” template in MRIStudio). Whole-brain fiber tracking was then performed to compute all of the streamlines in the brain—with fractional anisotropy (FA) > 0.15 and deviation angle < 50°—before implementing a multi-ROI approach (i.e., using the “Cut” operation in DTIStudio) to identify subsets of these entering or passing through pairs of nodes in each functionally-connected brain network (Shirer et al., 2012).

Due to the complexity and number of image processing steps necessary to generate normalized fiber tracts, our multi-stage DTI analysis pipeline made use of several different programs (Figure 1). Initial preprocessing and tensor fitting were performed with CATNAP *(Coregistration, Adjustment, and Tensor-solving, a Nicely Automated Program; http://iacl.ece.jhu.edu/~bennett/catnap/, Johns Hopkins University School of Medicine, Baltimore, Maryland, USA*), which applied a 12-parameter affine registration to: (1) coregister the diffusion-weighted and mean *b* = 0 s/mm^2^ images, (2) correct for motion and eddy current distortions, and (3) reorient the gradient direction for each diffusion-weighted image before generating the six tensor images (Landman et al., 2007). Brain extraction (or “skull stripping”) was then performed using a two-step procedure, whereby subject-specific brain masks were generated in SPM8 *(http://www.fil.ion.ucl.ac.uk/spm/software/spm8/, Wellcome Trust Centre for Neuroimaging, London, UK*) using the New Segment tool, and these were then manually refined using the ROIEditor toolbox in MRIStudio *(https://www.mristudio.org/, Johns Hopkins University School of Medicine, Baltimore, Maryland, USA)*. The coregistered and skull-stripped mean *b* = 0 s/mm^2^ images for each subject were then normalized to the “*JHU_MNI_SS_b0_ss”* template (Mori et al., 2008) in Montreal Neurological Institute (MNI) coordinate space (Mazziotta et al., 1995). This was implemented using the DiffeoMap toolbox in MRIStudio to carry out a 12-parameter affine (linear) transformation, followed by high-dimensional, non-linear warping with the large deformation diffeomorphic metric mapping (LDDMM) algorithm (Beg et al., 2005). The LDDMM analysis was performed with cascading elasticity (i.e., alpha values of 0.01, 0.005, and 0.002) to allow increasingly pliable deformations, as previously reported (Ceritoglu et al., 2009).

Each subjects' tensor images were warped to normalized ICBM space (Mazziotta et al., 1995) by applying the overall Kimap (linear affine + non-linear LDDMM) transformation, as previously described (Ceritoglu et al., 2009). This approach has previously been shown to compensate for susceptibility-induced B_0_ distortions (Huang et al., 2008); and, importantly, as long as the tensors are reoriented appropriately during the normalization procedure—as described by Alexander et al. (2001), Jones et al. (2002), and Xu et al. (2003)—fiber tracking can be performed for each subject in standard space. In this way, deterministic tractography was performed using a single-tensor model via the DTIStudio toolbox (Jiang et al., 2006) within MRIStudio, where white matter streamlines were identified using the Fiber Association by Continuous Tracking (FACT) algorithm and an exhaustive search approach (Mori et al., 1999; Xue et al., 1999). Tracking was initiated from a single seed located at the center of each voxel with a fractional anisotropy (FA) value greater than 0.15 and continued until FA fell below 0.15 or the deviation angle between adjacent vectors exceeded 50°, as previously reported (Yeatman et al., 2011). These values were chosen to be slightly more liberal than the default DTIStudio thresholds (FA > 0.2 and tract-turning angle < 40°) in order to: (1) ensure that fiber tracking would penetrate into cortical or sub-cortical gray matter regions, and (2) include streamlines with slightly higher deviation angles^1^.

A multi-ROI approach was then used to identify particular tracts between nodes of interest from the normalized, whole-brain tractography data. However, in order to first confirm the sensitivity and reliability of our image processing and tractography pipeline, we initially sought to examine a well-established white matter connection. Two Brodmann areas—i.e., left BA22 and left BA44, as defined in the Talairach Daemon (TD-ICBM Human Atlas) within the SPM8 WFU_PickAtlas Toolbox *(http://fmri.wfubmc.edu/software/pickatlas, Wake Forest University, Winston-Salem, NC*) (Lancaster et al., 2000; Maldjian et al., 2003)—were used to validate our tractography approach via the ability to measure streamlines along the putative left arcuate fasciculus. Because the two BA masks were restricted to the cortical sheet, and were not dilated to penetrate deeper into adjacent white matter regions, it should be noted that this constitutes a more rigorous test of our tractography method than the subsequent functionally-defined ROIs (which were generally larger and often descended further into the borders of the white matter). Nevertheless, despite this apparent handicap: (1) tractography streamlines were still observed between the left BA22 and left BA44 in the vast majority (27 out of 32) of subjects, and (2) the resulting group probability map demonstrated that (with rare exception) the topology of these fibers corresponded to the left arcuate fasciculus, as expected (Supplementary Figure 1).

After validating our preprocessing pipeline and deterministic tractography parameters with a known anatomical connection, we then employed the same methods in a more exploratory manner. Specifically, ROIs for each of six networks—including the dorsal and ventral Default Mode Networks (dDMN and vDMN) (Supplementary Videos 1, 2); the left and right Executive Control Networks (lECN and rECN) (Supplementary Videos 3, 4); and the anterior and posterior Salience Networks (aSN and pSN) (Supplementary Videos 5, 6)—were defined *a priori* using pre-existing atlases of functionally-connected brain networks *(http://findlab.stanford.edu/functional_ROIs, Stanford University, Palo Alto, CA)* (Shirer et al., 2012)^2^. ROI-to-ROI contingencies were then generated for every pair of nodes within each network, and these contingency maps were applied to each subject's whole-brain tractography data using the “Cut” operation in DTIStudio to identify the FACT streamlines running between both network nodes specified in the ROI-to-ROI contingencies^3^. Therefore, while no minimum length threshold was specified in the tractography analysis, the length of each streamline must (by definition) have been greater than or equal to the distance between each pair of nodes in the ROI-to-ROI analysis. In this way, subsets of tracts were identified for each subject that: (1) met the deterministic tractography criteria and (2) entered or passed through both nodes for each possible ROI-to-ROI pair (i.e., within each of the six networks investigated).

Since the dDMN consists of 9 nodes (36 ROI-to-ROI combinations), the vDMN consists of 10 nodes (45 ROI-to-ROI combinations), the lECN consists of 6 nodes (15 ROI-to-ROI combinations), the rECN consists of 6 nodes (15 ROI-to-ROI combinations), the aSN consists of 7 nodes (21 ROI-to-ROI combinations), and the pSN consists of 12 nodes (66 ROI-to-ROI combinations), 198 ROI-to-ROI contingencies were assessed for each of the 32 subjects—for a total of 6336 tractography analyses. For each of these analyses, the data were visually inspected to identify subjects for whom continuous streamlines were present for each ROI-to-ROI contingency and any/all streamlines were saved as binary maps (in normalized space). Group probability maps for each of the 198 functionally-defined tracts were then computed by combining (i.e., adding together) the binary maps for each of the subjects for a given ROI-to-ROI contingency and then dividing by 32 (i.e., the number of subjects). Thus, image intensities for each of the group probability maps have limits of 0 and 1 (i.e., for voxels in which no subjects or all 32 subjects exhibited a streamline, respectively).

For visualization purposes, 3D projections of the network nodes and white matter probability maps were constructed using the Volume and Volume Rendering tools in 3D Slicer *(http://www.slicer.org, Brigham and Women's Hospital, Boston, MA*) (Fedorov et al., 2012). To achieve this, the network nodes and their corresponding functionally-defined, probabilistic white matter tract(s) were first rendered using the NCI GPU Ray Casting method, and the resulting 3D reconstruction was then overlaid on an anatomical template image (which was either the “JHU_MNI_SS_T1” image from MRIStudio for all of the white matter tracts or the “avg152T1” image from SPM8 for the network nodes).

Finally, in order to demonstrate how our atlases might be used in future studies to infer relationships between white matter structure within each of these networks and other variables of interest (e.g., age, cognitive test scores, disease progression, etc.), we created a toy example by taking age as an independent variable and then performing two different types of analyses with subjects' normalized FA images. In the first type of analysis, the FA images were smoothed with a 4 mm FWHM 3D smoothing kernel and a second-level (i.e., between subjects), voxel-wise general linear model analysis was performed to identify regions where FA was positively or negatively associated with age (FDR-adjusted *p* < 0.05). White matter regions identified as having significant correlations with age were then compared to each of the functionally-defined white matter networks to determine the amount of spatial overlap between the voxel-wise statistical maps and each white matter network^4^. The second type of analysis was more of a conventional ROI-based approach, where the mean FA values were extracted from each white matter network across all 32 subjects and used to perform linear correlations between FA and age for each network.

## Results

**Figure 2:**
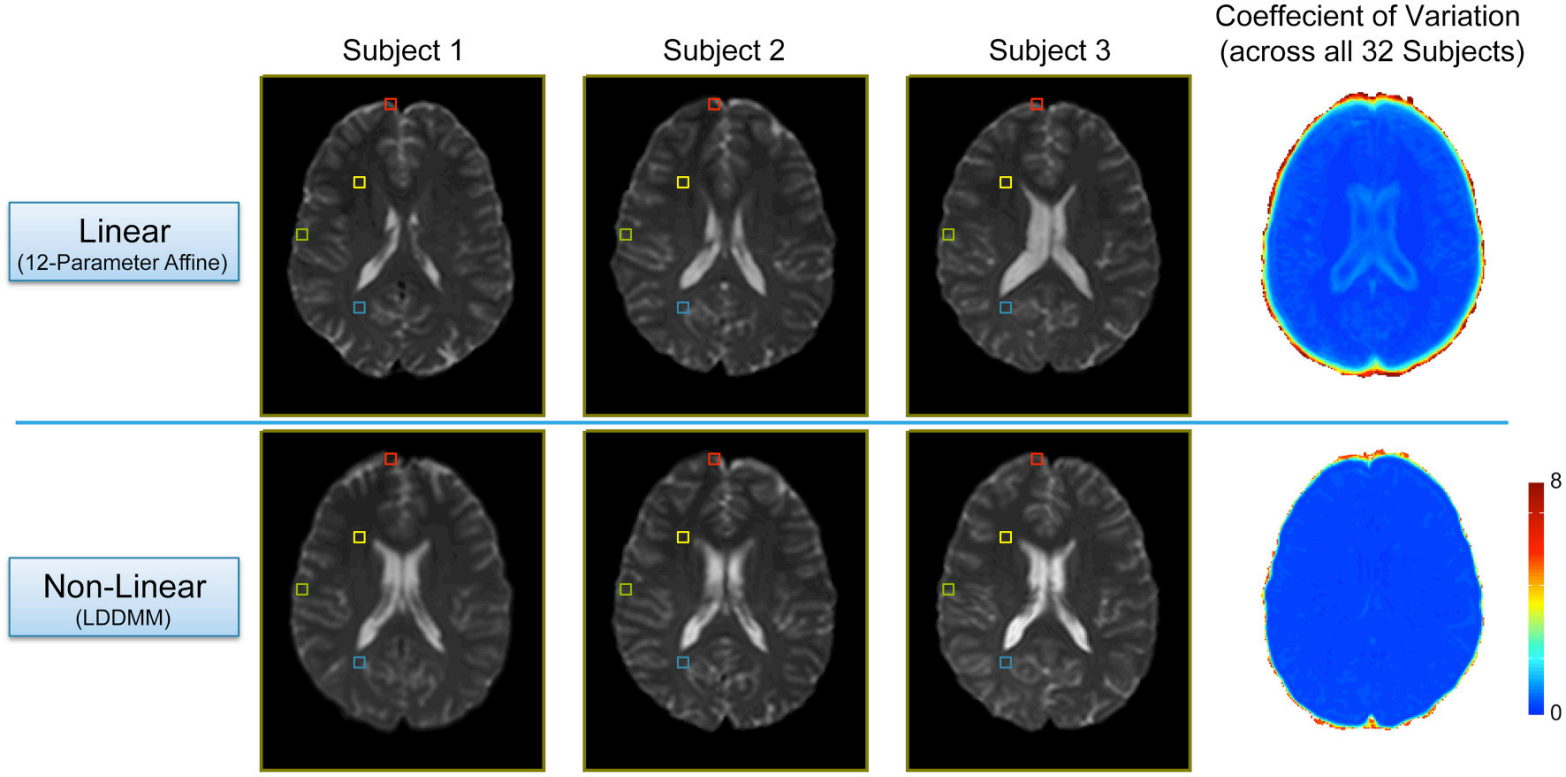
**Intermediate and final results of the two-stage, non-linear normalization procedure**. **Top Row:** Mid-axial slices from three representative subjects (i.e., the first three sorted by first initial) after the 12 parameter linear normalization (i.e., Automated Image Registration in DiffeoMap), as well as the coefficient of variation (COV) image across all 32 subjects showing good alignment and overall scaling, but large subcortical differences between subjects. **Bottom Row:** Both the individual images, as well as the COV image show marked improvement after the subsequent non-linear (LDDMM) normalization step with three phases of cascading elasticity. This highlights the need for future investigators to use the same (or similar) non-linear normalization approaches when interpreting their quantitative white matter imaging findings in the context of our group probability maps.

In order to evaluate the efficacy of the two-stage linear (12-parameter affine) and non-linear (LDDMM) normalization approach, we compared each subject's warped mean *b* = 0 s/mm^2^ image (i.e., the average of all five *b* = 0 s/mm^2^ images acquired in the DTI pulse sequence) and calculated coefficient of variation maps across all 32 subjects after each step (Figure 2). As expected, the linear normalization step was effective for overall scaling and cortical alignment, but large inter-subject differences remained throughout subcortical regions (Figure 2; Top Row), most notably in the deep, periventricular white matter. However, the subsequent non-linear (LDDMM) normalization step corrected these inter-subject variations, producing highly consistent subcortical alignment across subjects (Figure 2; Bottom Row). Although it required substantially more time and effort, the efficacy of the high-dimensional, non-linear normalization approach was significant for at least three reasons. By warping each subject's tensor images to normalized space, it: (1) enabled us to make use of the previously published ROIs from each network (to create all of the ROI-to-ROI contingencies); (2) allowed us to combine tract information across subjects (to create the probabilistic atlases for each tract); and (3) will allow future studies to either extract quantitative measures of white matter microstructure from these regions to make cross-subject comparisons or assess the amount of overlap compared to voxel-wise studies. However, as shown in Figure 2, future studies aiming to use our normalized atlases for quantitative analyses must implement similar high-dimensional, non-linear normalizations in their image processing pipelines, and not simply rely on “standard” linear normalizations.

**Figure 3:**
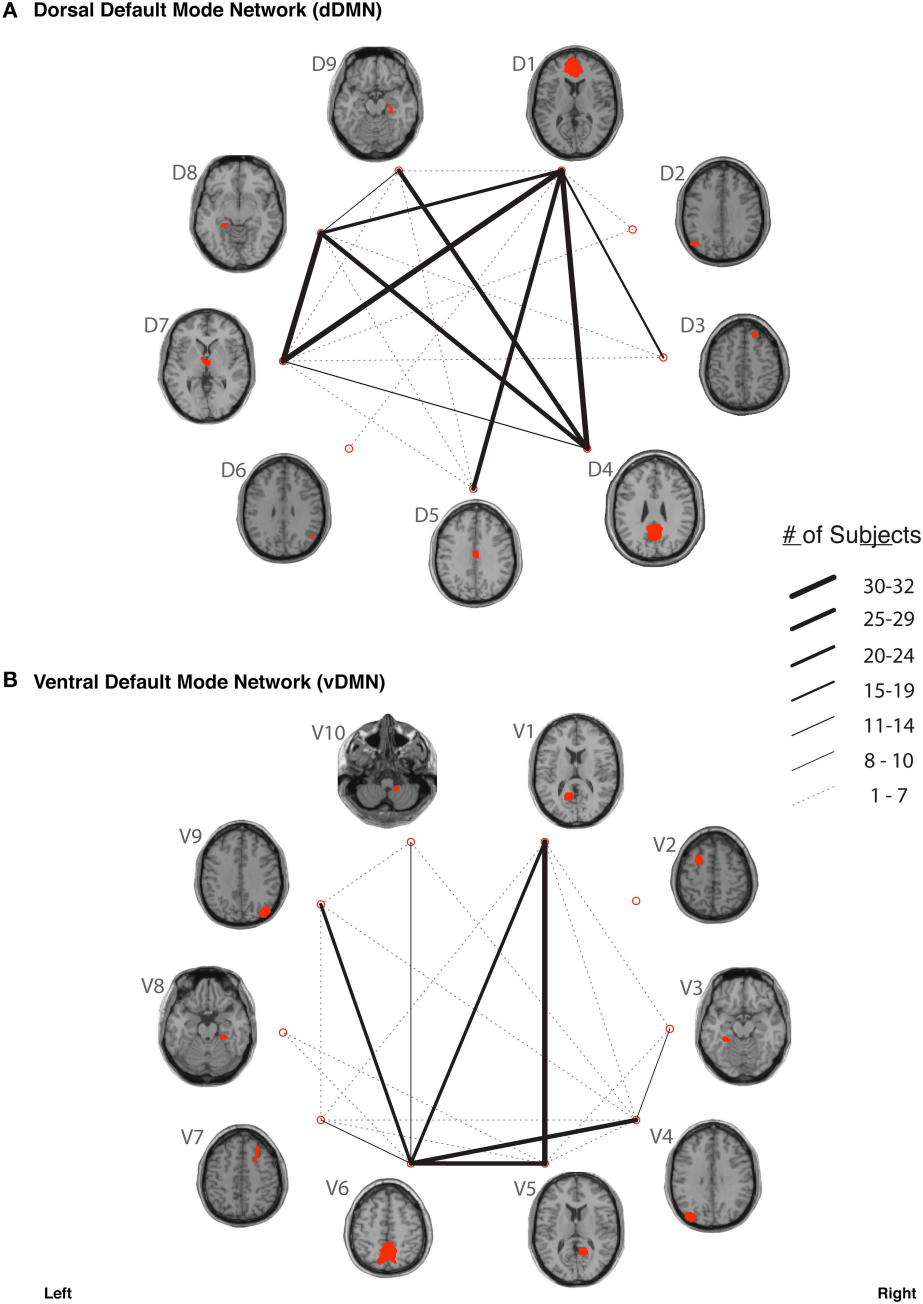
**The connection counts for each functionally-defined white matter tract in (A) the dorsal Default Mode Network (dDMN) and (B) the ventral Default Mode Network (vDMN)**. The nodes within each network (Shirer et al., 2012) are shown on axial brain slices (at their center-of-mass) in red, and the connection counts for each tract (i.e., the number of subjects with tractography streamlines identified between each ROI-to-ROI pair) are represented by the weight of the lines connecting the respective nodes.

**Figure 4:**
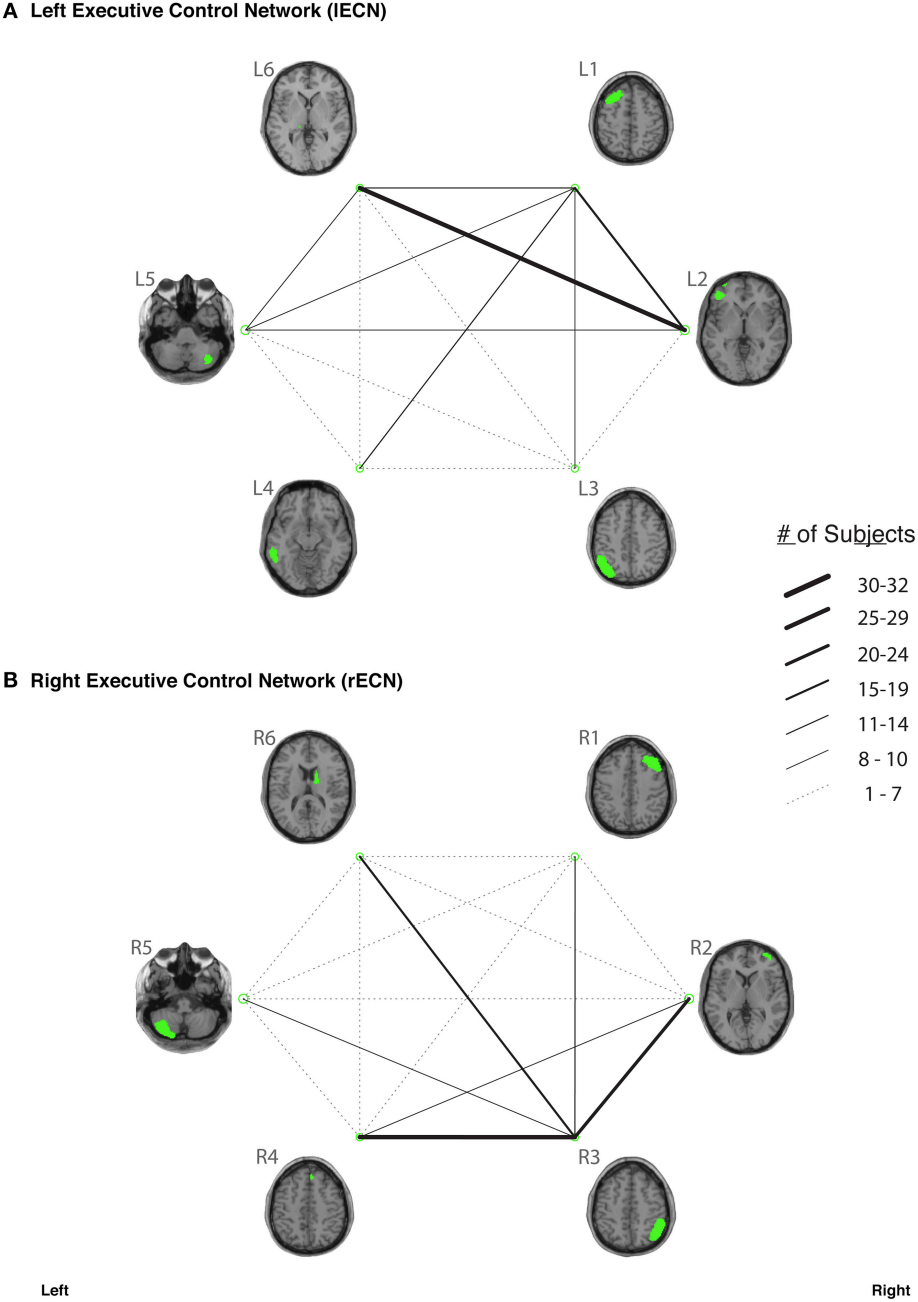
**The connection counts for each functionally-defined white matter tract in (A) the left Executive Control Network (lECN) and (B) the right Executive Control Network (rECN)**. The nodes within each network (Shirer et al., 2012) are shown on axial brain slices (at their center-of-mass) in green, and the connection counts for each tract (i.e., the number of subjects with tractography streamlines identified between each ROI-to-ROI pair) are represented by the weight of the lines connecting the respective nodes.

**Figure 5:**
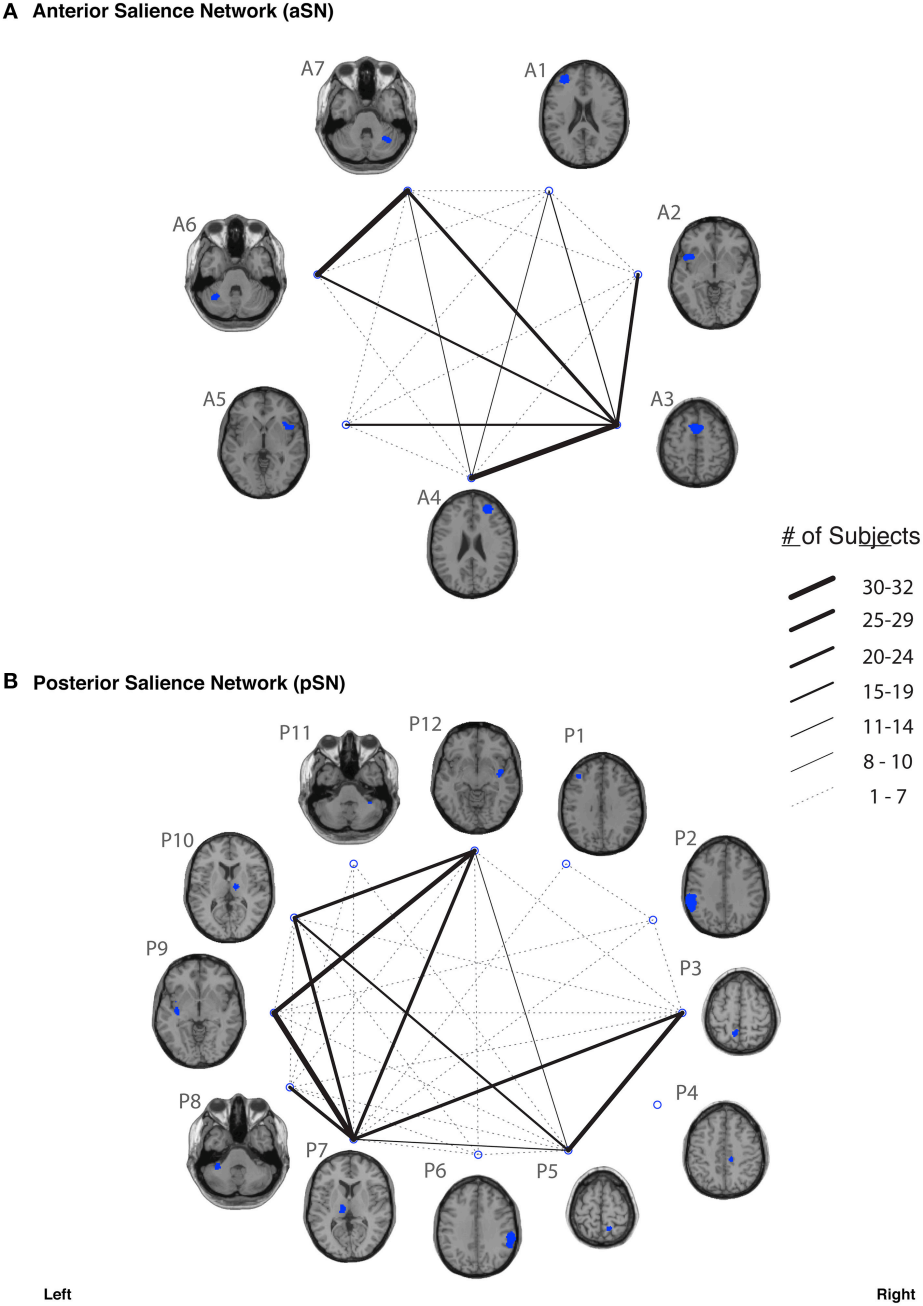
**The connection counts for each functionally-defined white matter tract in (A) anterior Salience Network (aSN) and (B) the posterior Salience Network (pSN)**. The nodes within each network (Shirer et al., 2012) are shown on axial brain slices (at their center-of-mass) in blue, and the connection counts for each tract (i.e., the number of subjects with tractography streamlines identified between each ROI-to-ROI pair) are represented by the weight of the lines connecting the respective nodes.

In our study, fiber tracking was used to search for all white matter connections between the nodes within each of six functionally-defined brain networks. Of the 198 separate ROI-to-ROI contingencies, some had streamlines that were commonly identified across subjects, while others did not. The “connection counts” (Zhang et al., 2010)—i.e., the number of subjects exhibiting at least one streamline—for each ROI-to-ROI pair are depicted in Figure 3 (dDMN and vDMN), Figure 4 (lECN and rECN), and Figure 5 (aSN and pSN). Interestingly, many of the ROIs with high connection counts to multiple other regions have previously been noted to have the highest degrees of white matter interconnectivity (Van den Heuvel and Sporns, 2011). These regions include: D1, D4, D8, and D7 in the dDMN (corresponding to the anterior cingulate/medial prefrontal cortex, posterior cingulate/precuneus, left parahippocampal gyrus and thalamus, respectively); V1, V5, and V6 in the vDMN (corresponding to the left posterior cingulate, right posterior cingulate, and precuneus, respectively); R3 in the rECN (corresponding to the inferior/superior parietal lobule); A3 in the aSN (corresponding to the anterior cingulate); and P7, P9, P10, and P12 in the pSN (corresponding to the left thalamus, left insula/claustrum, right thalamus, and right insula/claustrum, respectively). However, to rule out the possibility that these connection counts were simply related to the distance between ROIs (e.g., that proximal ROI pairs produced systematically higher connection counts than distal ROI pairs), the connection counts between network nodes were also depicted after applying multidimensional scaling^5^ to separate nodes according to the Euclidean distance between each node's center of mass (Supplementary Figure 2). The large number of tracts with high connection counts, including many long-range connections, suggests: (1) that each of these functionally-connected networks has a highly organized set of underlying white matter structural connections, and (2) that the tractography results are fairly robust across subjects. Moreover, in order to minimize the number of spurious fiber tracts included in the atlases, all subsequent analyses (including group probability map calculations) were limited to tracts with connection counts of at least 8/32 (i.e., tracts in which one or more streamlines were identified in at least ¼ of the subjects).

**Figure 6:**
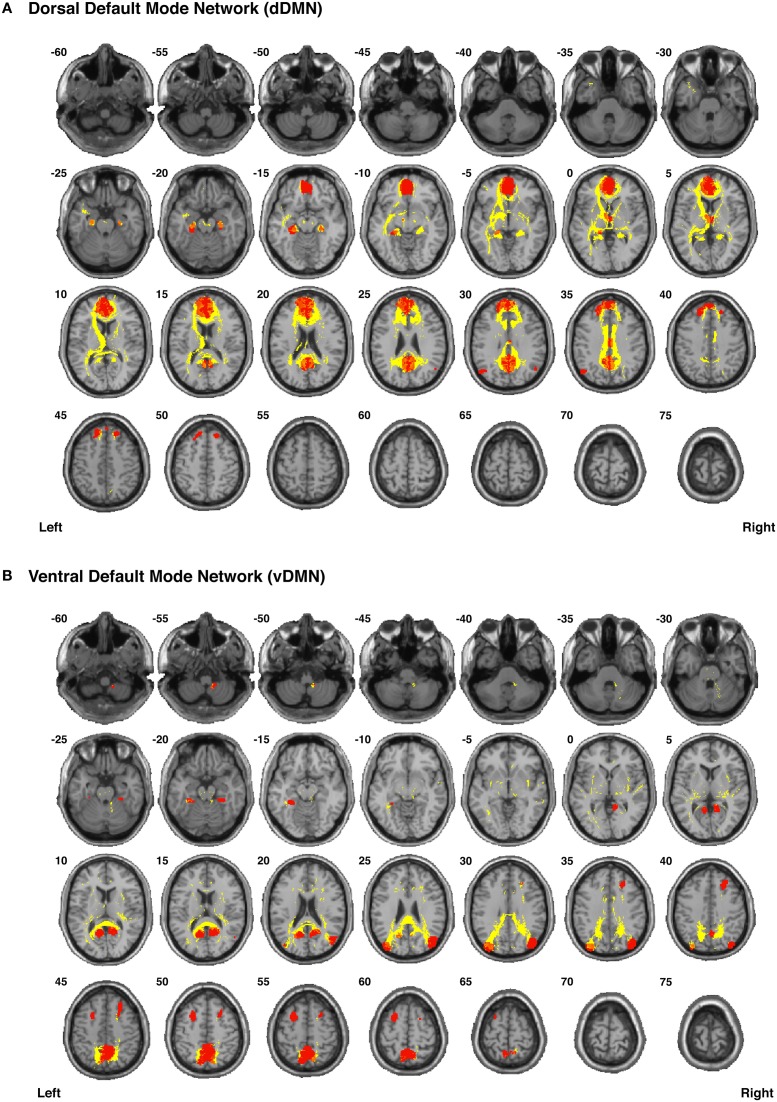
**Binary masks of all of the nodes (red) and all of the functionally-defined group probability maps (yellow) in (A) the dorsal Default Mode Network (dDMN) and (B) the ventral Default Mode Network (vDMN) to show their spatial extents and locations**. See Supplementary Videos for 3D renderings of the group probability maps of each individual tract (Supplementary Videos 7–25), as well as the overall networks (Supplementary Videos 60–61) in greater detail.

**Figure 7:**
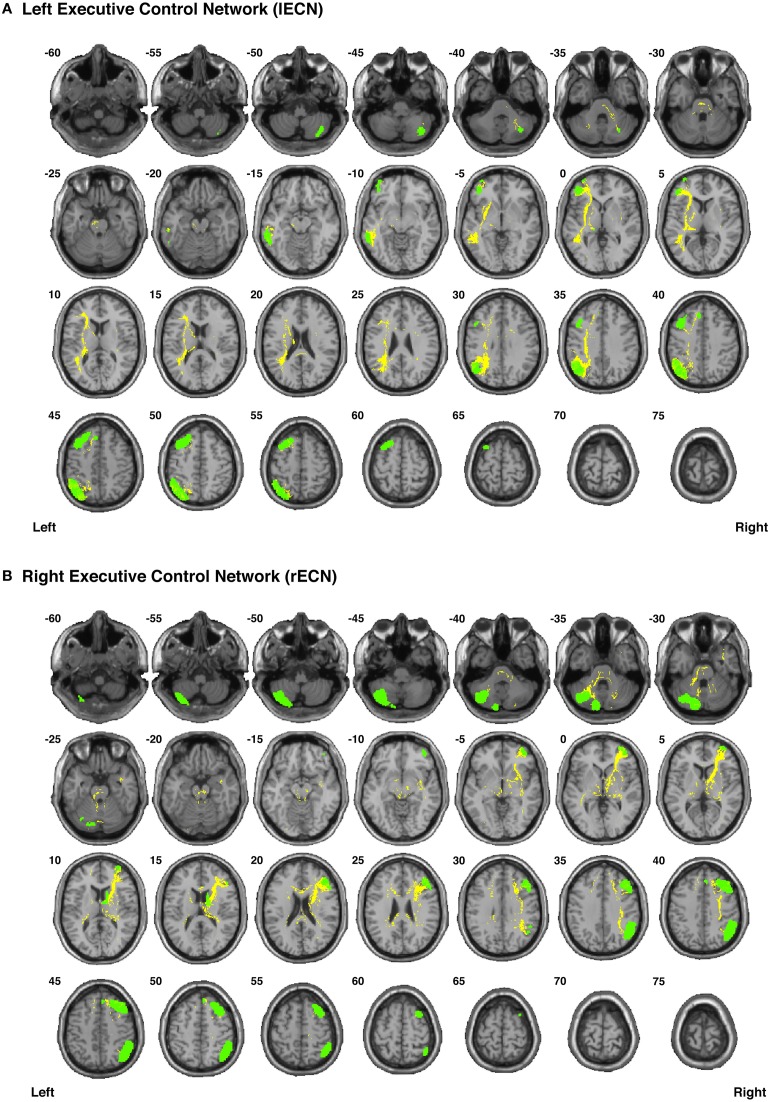
**Binary masks of all of the nodes (green) and all of the functionally-defined group probability maps (yellow) in (A) the left Executive Control Network (lECN) and (B) the right Executive Control Network (rECN) to show their spatial extents and locations**. See Supplementary Videos for 3D renderings of the group probability maps of each individual tract (Supplementary Videos 26–39), as well as the overall networks in greater detail (Supplementary Videos 62–63).

**Figure 8:**
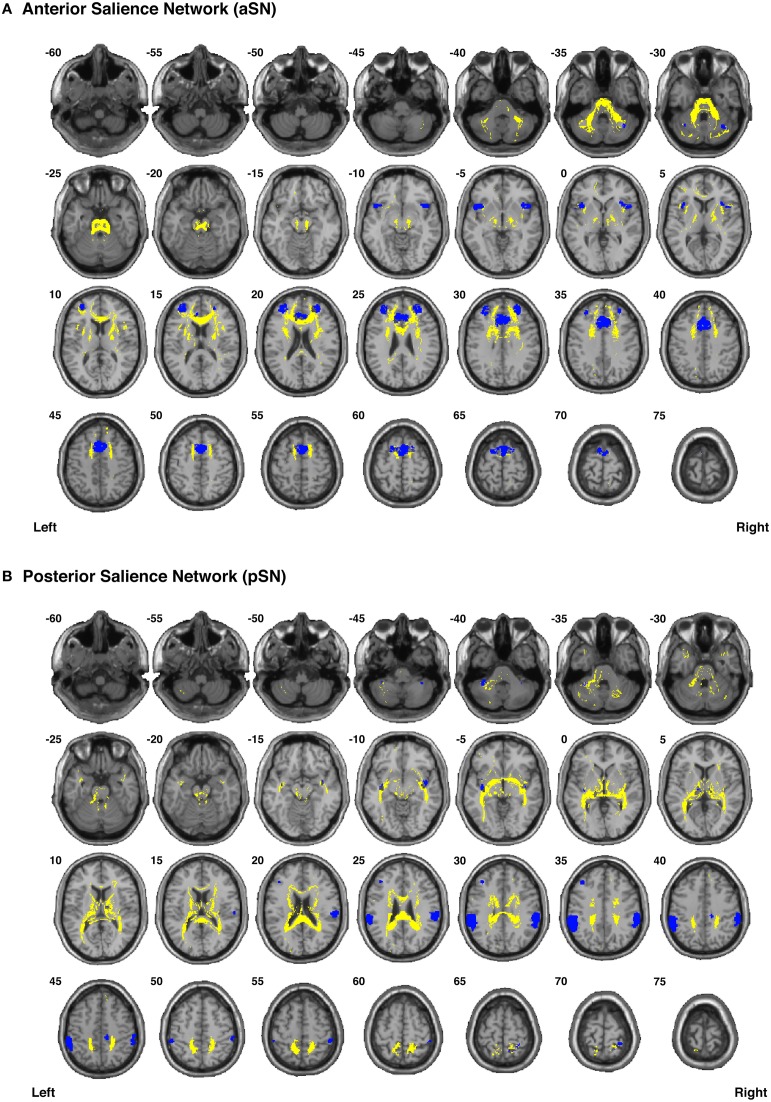
**Binary masks of all of the nodes (blue) and all of the functionally-defined group probability maps (yellow) in (A) the anterior Salience Network (aSN) and (B) the posterior Salience Network (pSN) to show their spatial extents and locations**. See Supplementary Videos for 3D renderings of the group probability maps of each individual tract (Supplementary Videos 40–59), as well as the overall networks in greater detail (Supplementary Videos 64–65).

Our functionally-defined white matter tracts, along with the corresponding nodes from each network, are shown as binary masks in Figure 6 (dDMN and vDMN), Figure 7 (lECN and rECN), and Figure 8 (aSN and pSN); however, the group probability maps for each tract are depicted in Supplementary Videos 7–59, and the combined group probability maps for each overall network (i.e., a superposition of all individual tracts within each network) are displayed in Supplementary Videos 60–65. Each of these probabilistic maps reflects the common and reproducible tract trajectories across subjects, and can be thresholded according to the amount of desired between-subject overlap (e.g., thresholding an image at 0.25 will show only those regions where at least ¼ of the subjects' streamlines spatially overlap, etc.). Although it has been previously discussed (Aron et al., 2007; Zhang et al., 2010), it is perhaps worth reiterating here that the group probability maps are more conservative than the raw connection counts. This stems from the fact that connection counts only represent the number of subjects who had at least one continuous streamline between two regions (regardless of the spatial locations of the voxels comprising each streamline), whereas the group probability maps represent the proportion of subjects who have overlapping streamlines that are in exactly the same spatial location. Therefore, owing to different streamline trajectories across subjects, values below 0.25 are possible in the group probability maps, despite the requirement for each of them to have had a connection count greater than or equal to 8/32 (i.e., in order to eliminate biologically spurious or unlikely tracts).

It is also important to note that while the JHU_MNI templates distributed with the MRIStudio packages (i.e., DTIStudio, ROIEditor, and DiffeoMap) are correctly normalized to the MNI template, they are spatially offset compared to the SPM8 template. Therefore, we have coregistered and compiled all of our group probability maps (i.e., for each individual tract, as well as all of the tracts in each network) in both coordinate systems so that they can be conveniently used with either SPM or MRIStudio in future studies. A folder containing all group probability maps (i.e., for each individual tract and each network as a whole), as well as the Supplementary Videos showing their 3D trajectories, can be freely downloaded from the NITRC website (www.nitrc.org/projects/uofm_jhu_atlas).

The total white matter volume of each network (in normalized MNI space) is shown in Supplementary Figure 3. Of the six networks, the largest white matter volume was occupied by the dDMN, followed by the pSN, aSN, vDMN, and then the lECN and rECN (which had almost identical volumes). Since each dataset was resampled and interpolated during the two-stage non-linear normalization procedure—which preceded all of the subsequent analyses (including tractography)—the group probability maps and volumetric analyses both have had the benefit of being calculated with 1 mm isotropic resolution. Thus, the volume of each functionally-defined white matter network was calculated by creating an overall mask of the tract-level group probability maps within each network (shown in Figures 6–8) and simply counting the number of voxels in the mask without placing any additional constraints (other than the deterministic thresholds, ROI-to-ROI contingencies and the ≥8/32 connection counts used to originally create the group probability maps). Using these same overall network masks, we were then able to calculate the amount of spatial overlap between the white matter regions assigned to each network and report these as actual volumes (Supplementary Figure 4A) or normalized ratios, compared to the to the size of each network (Supplementary Figure 4B). Perhaps not surprisingly, the largest overlap in terms of absolute volume was observed between the two largest network masks (i.e., the dDMN vs. pSN), followed by the dDMN vs. aSN, dDMN vs. vDMN, etc. However, in terms of relative overlap (proportional to the size of each network), the largest overlaps were between the vDMN vs. dDMN, followed by the pSN vs. dDMN, aSN vs. dDMN, etc.

**Figure 9:**
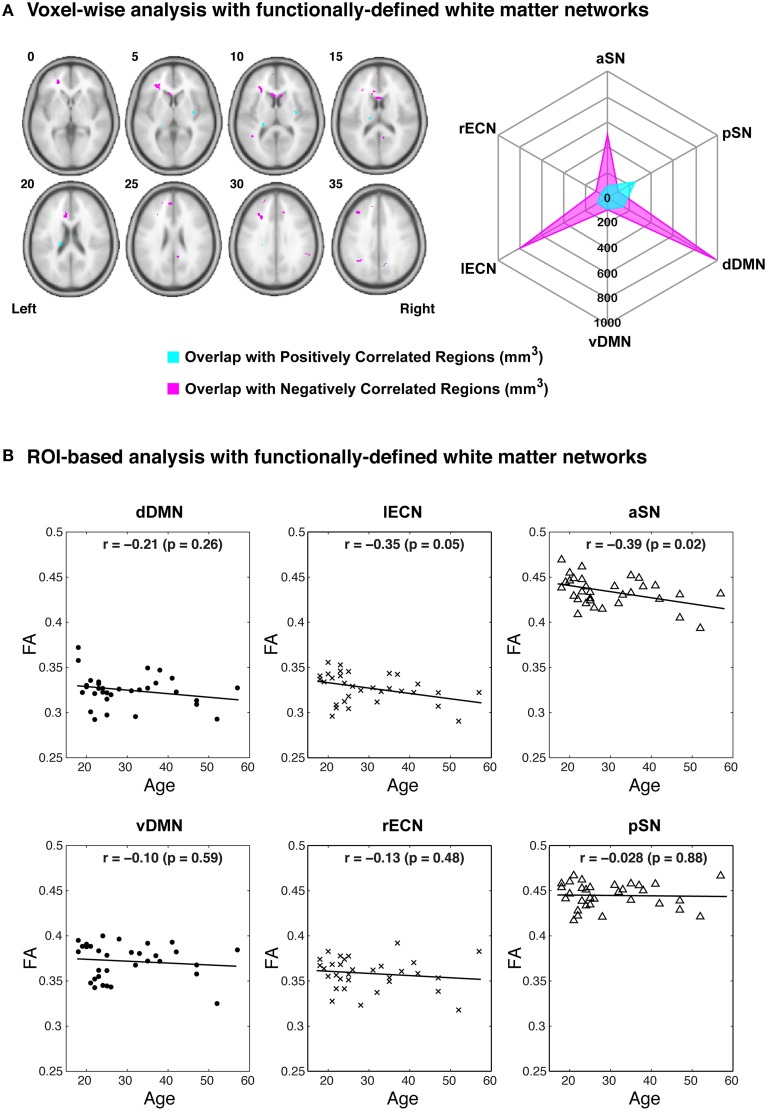
**Examples of potential voxel-wise and ROI-based analyses using the functionally-defined white matter atlases described above**. **(A)** After performing a standard voxel-wise analysis to identify any white matter regions where FA is positively (magenta) or negatively (cyan) correlated with age (FDR-adjusted *p* < 0.05; left panel), the regions can be compared to each of the white matter network masks to determine the amount of spatial overlap (e.g., overlap volume in mm^3^; right panel). In this way, the negative voxel-wise correlations between age and FA can be ascribed primarily to three functionally-defined white matter networks (i.e., the dDMN, lECN, and aSN). **(B)** Alternatively, the relationships between FA and age can be investigated using a standard ROI-based approach (i.e., to calculate the mean FA within each white matter network for each subject). When analyzed in this way, it appears that higher age in our sample population is associated with decreased FA throughout the lECN (*p* = 0.05) and aSN (*p* = 0.02).

Examining the FA values for each white matter network (Table 1) revealed that the dDMN and lECN were significantly lower (*p* < 0.001) compared to the average across all networks; the rECN displayed a trend toward lower FA values (*p* = 0.047, which is not significant after correcting for multiple comparisons); and the aSN and pSN had significantly higher FA values (*p* < 0.001). Moreover, the FA images were also used to demonstrate both types of analyses that our white matter atlases might help to address in future studies. After calculating statistical parametric maps to examine regional FA changes related to age (or any other hypothesis-driven independent variable) and creating thresholded masks with an FDR-adjusted *p* < 0.05 (Figure 9A; left panel), the amount of overlap can be assessed with each white matter network. In our sample, age-related FA differences were predominantly located in the white matter regions nominally ascribed to the dDMN, lECN, and aSN, as opposed to the other three networks, which exhibited very little overlap (Figure 9A; right panel). The ROI-based analyses (Figure 9B), where FA values were extracted from each white matter network mask and then regressed with age for each subject, showed similar (albeit arguably less powerful) results. In this case, the two networks that exhibited trending negative associations between overall network FA and age were the lECN (*p* < 0.05) and the aSN (*p* < 0.02). Perhaps not surprisingly, the voxel-wise and ROI-based analyses identified the same two or three networks exhibiting the strongest negative associations between age and FA; and neither approach found significant positive associations (between FA and age) in any network.

**Table 1:** **Mean and standard deviation of the FA values within each functionally-defined white matter network (i.e., across all 32 subjects), as well as the statistical significance (***p***-value) of the difference (i.e., compared to the FA values obtained across all six networks in a two-tailed ***t***-test)**.

	**dDMN**	**vDMN**	**lECN**	**rECN**	**aSN**	**pSN**	**All Networks**
Mean FA	0.325	0.372	0.327	0.358	0.434	0.445	0.377
Std	0.018	0.019	0.016	0.018	0.016	0.014	0.050
*p*-value	< 0.001	0.59	< 0.001	0.047	< 0.001	< 0.001	–

*The dDMN, lECN and rECN appear to have significantly lower FA values compared to the average across all networks, while the aSN and pSN appear to have significantly higher FA values*.

## Discussion

### General discussion

Anatomically-defined white matter atlases and white matter probability maps have been created in the past by other groups, but to the best of our knowledge, this is perhaps the most comprehensive set of functionally-defined probabilistic white matter atlases reported to date. Given what we now know about the architecture of the brain and its organization into intrinsic, distributed networks, we anticipate that our atlases will be a useful tool in future studies aiming to assess white matter microstructure within the Default Mode, Executive Control and Salience Networks and the ability to relate structural changes within these networks to clinical deficits, cognitive performance, functional connectivity, etc. As demonstrated, they can be used in combination with: (1) voxel-wise analyses (e.g., linear regressions between DTI or any other white matter imaging data and any set of independent variables) to assess the amount of overlap with each probabilistic atlas—i.e., allowing the voxel-wise changes to be ascribed to the white matter regions underlying a particular functional network or group of networks (e.g., Figure 9A); or (2) ROI-based analyses to examine relationships between structural measures throughout an entire functionally-defined tract or network (e.g., Figure 9B). Moreover, the current atlases (or more likely the individual group probability maps of the component tracts) could theoretically be used in conjunction with other novel analysis methods that extract diffusion metrics along white matter pathways (c.f., Walsh et al., 2011; Colby et al., 2012; Yeatman et al., 2012).

It should be noted that other groups have performed somewhat similar fMRI-guided DTI analyses within portions of the Executive Control and Default Mode Networks; however, to the best of our knowledge, none have been as thorough in their analysis nor as comprehensive in terms of the number of nodes or subjects studied. For example, one earlier study (Aron et al., 2007) created functionally-defined white matter maps between three pre-determined executive regions—namely the right inferior frontal cortex (IFC), subthalamic nucleus (STN), and pre-supplementary motor area (preSMA)—and showed that the tractography data were consistent with fMRI responses elicited by a cognitive stop-signal task. However, due to the specific hypotheses of this study, only the white matter connections between these select few ROIs were investigated across 10 subjects. Similarly, another study (Greicius et al., 2009) has examined a select number of structural connections between three sets of nodes in the DMN—specifically the bilateral connections between the medial prefrontal cortices (mPFC), posterior cingulate/retrosplenial cortices (PCC/RSC), and middle temporal lobes (MTL)—showing that two out of their three contingencies yielded robust tractrography results across 20 healthy subjects. However, due to the specific hypotheses of the study and certain methodological limitations at the time, tracts between more/other nodes were not examined. Alternatively, another recent study has implemented a sophisticated fiber-tracking technique to measure structural connectivity throughout the entire cortex in an observer-independent manner and compared these findings to whole-brain, voxel-wise functional connectivity matrices (Horn et al., 2014). Their analyses revealed that certain areas within the DMN showed the highest agreement between structural and functional connectivity, suggesting that this network may have the most direct structural connections—an observation that appears to be partially supported by the relatively high connection counts and overall size of the dDMN and vDMN borne out in our tractography data.

Since the inputs and outputs of any given brain region determine both the information available to it and its ability to influence other regions, a comprehensive description of the structural connections within the human brain—generally referred to as the “human connectome”—is central to systems and cognitive neuroscience (Sporns et al., 2005; Van Essen and Ugurbil, 2012). In this regard, the stereotaxic white matter probability maps generated in the current study form a kind of “functionally-defined connectome” and are expected to have widespread utility. The recent trend within systems and cognitive neuroscience regarding intrinsic brain networks has (at least to date) been primarily dominated by studies focusing on functional connectivity changes, with far fewer studies investigating white matter connectivity. This disparity is almost certainly related to the fact that functional connectivity capabilities (i.e., for ROI- and/or ICA-based resting state fMRI analysis) are now available in every major fMRI analysis package, and there are a growing number of network-based atlases, like the ones reported by Shirer et al. *(http://findlab.stanford.edu/functional_ROIs, Stanford University, Palo Alto, CA*), to facilitate these analyses. Therefore, it is our hope that the white matter atlases reported here will act as a compliment to the Stanford group's functional connectivity atlases, and that they will be used to facilitate future studies examining white matter structural connectivity within these networks.

Finally, in addition to basic research applications, these atlases could potentially have certain translational or clinical applications. For example, the “clinico-radiological paradox” (Barkhof, 2002) is a well-known phenomenon among patients with white-matter disorders (including Multiple Sclerosis, etc.), where the associations between clinical symptoms and common radiological markers (e.g., lesion volume, number of lesions, etc.) are typically quite poor. However, preliminary evidence suggests that this phenomenon has to do with intersubject differences in lesion locations (Hackmack et al., 2012)—where the degree of damage to a particular functionally-defined network (including its underlying white matter) would be expected to cause specific clinical symptoms related to the role of that network. Therefore, in future studies, lesion locations could be compared to our functionally-defined white matter atlases to test this hypothesis; and if confirmed, they could perhaps be used in a diagnostic and prognostic capacity. Furthermore, given the central nature of the networks investigated in the current study and their role in high-level cognition and executive function, our atlases could conceivably be inversely normalized into subject space and used in concert with task-related and/or resting-state fMRI (Lee et al., 2013) for the purpose of presurgical planning (e.g., prior to epileptic lobectomy or tumor resection) to minimize postoperative functional deficits.

### Structure-function relationships

The organization of neuronal connections throughout the CNS is thought to be specific at multiple levels, such that: (1) each brain region is connected to only a small subset of other regions, and (2) within any given cortical region, the afferent and efferent fibers are organized in precise, layer-specific patterns (Callaway, 2002). In the current work, we sought to study the long-range white matter pathways between functionally-connected cortical regions using DTI tractography, and to construct probabilistic atlases of these connections within previously defined functional networks. Although certain pairs of functional nodes were consistently connected by white matter streamlines in our analysis (Figures 3–5 and Supplementary Figure 2), there were several node pairs for which direct white matter connections were not commonly observed. This suggests that either there were underlying white matter connections that our tractography methods were unable to detect (see discussion of Type II errors in the Study Limitations below), or that not all of the nodes within each network are interconnected by direct white matter pathways.

Regarding the latter, it is interesting to note that our findings are consistent with a handful of previous reports. For example, early studies of structure-function relationships within single brain slices showed that regions with direct white matter connections tended have high levels of functional connectivity, but that the inverse was not necessarily true (Koch et al., 2002); and later studies measuring whole-brain structure-function correlations also concluded that robust structural connectivity was predictive of functional connectivity, but that strong functional connectivity did not reliably predict structural connectivity (Honey et al., 2009). The present findings therefore strengthen previous hypotheses that structural connections are predictive of functional connectivity measures, but that functional connectivity or network membership is not strictly predicated on direct structural connections, since strong functional connectivity may also exist between regions without direct anatomical connections (c.f., Honey et al., 2010).

One explanation for robust functional connectivity despite the absence of direct anatomical connections between every pair of nodes likely has to do with the ways in which constituent parts of these networks are interrelated or arranged (i.e., their “topology”). For example, a number of studies have demonstrated that structural and functional networks share many important topologic features, including: small-world properties, modularity, hierarchy, and the existence of highly connected hubs (for reviews, please see Bullmore and Sporns, 2009, 2012; Wang et al., 2015). In particular, small world networks—i.e., a type of mathematical graph in which most nodes in a network can be reached from every other through only a small number of steps—have been adopted as an attractive and parsimonious model for brain organization because they can support both segregated and distributed information processing, accommodate high dynamical complexity, and minimize wiring and communication costs (Bassett and Bullmore, 2006). By observing the connection counts between various ROIs in our analysis (Figures 3–5), it is evident that certain nodes (e.g., D1 and D4 in the dDMN, V6 in the vDMN, etc.) are highly structurally connected and are therefore well-positioned to serve as network hubs; while, on the other hand, certain nodes appear to be structurally disconnected or isolated from the rest of the network (e.g., D6 in the dDMN, V2 in the vDMN, etc.). These findings appear to correspond with previous studies of the DMN which have shown that precuneus/posterior cingulate regions (i.e., corresponding to nodes D4 in the dDMN and V6 in the vDMN) exhibit consistently high levels of functional connectivity with the rest of the nodes in the DMN, while nodes in the medial temporal lobes (i.e., corresponding to nodes V3 and V8 in the vDMN) have consistently weaker interactions with the rest of the nodes in the DMN (Fransson and Marrelec, 2008). Taken together, this tends to suggest that functional hubs within these networks are also structural hubs. However, it should be kept in mind that we did not examine any of the structural connections between nodes in different networks (e.g., between dDMN and vDMN nodes), and it is possible that nodes with little or no structural connectivity within each sub-network could have direct white matter connections to other regions within higher levels of the network (e.g., the larger DMN as a whole).

### Study limitations

In general, diffusion imaging has several advantages compared to alternative white matter staining, tracer and microscopy methods. It is non-invasive, can provide whole-brain coverage to allow 3D examination of intact networks, and is therefore the only *in vivo* technique to estimate fiber trajectories between distributed cortical regions in humans. Nonetheless, this technique does have limitations and therefore warrants a few caveats. Both our DTI data acquisition parameters and analysis pipeline were optimized in an attempt to avoid well-known pitfalls (c.f., Jones and Cercignani, 2010) that might otherwise reduce data quality or lead to spurious interpretations. However, even when DTI data are properly acquired and analyzed, it is worth bearing in mind that these signals and their subsequent interpretation are ultimately derived from the diffusion characteristics of water molecules as they interact with their local environment (Beaulieu, 2002; Mori and Zhang, 2006). While previous studies have shown that DTI data can be highly correlated with microscopic staining and tracer techniques, correlations with these gold-standard methods depend on both the analysis parameters and the regions investigated (c.f., Johansen-Berg and Rushworth, 2009). Moreover, even under ideal conditions, DTI streamlines: (1) cannot necessarily differentiate myelinated vs. unmyelinated vs. demyelinated fibers (Beaulieu, 2002), (2) do not distinguish the anterograde vs. retrograde directionality of these fibers (Mori and Zhang, 2006), (3) may not discriminate between monosynaptic and polysynaptic connections (Johansen-Berg and Rushworth, 2009), and (4) should not be used in isolation (i.e., without supporting data or hypotheses) to draw conclusions about the degree of myelination, fiber/axon counts or “white matter integrity” (Jones et al., 2013).

Although high angular resolution diffusion imaging (HARDI) (Tuch et al., 2002), Q-ball imaging (Tuch, 2004), diffusion spectrum imaging (DSI) (Wedeen et al., 2005) and other more advanced diffusion MRI acquisition and analysis methods offer certain advantages over the more conventional DTI approach used here (e.g., their ability to deal, at least to some extent, with crossing fibers, etc.), it is important to bear in mind that all diffusion-based methods share many of the same fundamental limitations, and are still only surrogate markers of white matter microstructure and fiber orientation. The main difference is that while acquisition schemes with relatively few diffusion-encoding directions and low *b*-values have certain advantages (i.e., short acquisition times, less subject motion, and high signal-to-noise images), the analysis of such data are limited to relatively simple tensor-based models that are unable to resolve fiber crossings as well as more complex Q-space sampling approaches and reconstruction techniques (Daducci et al., 2014). However, one recent study comparing tractography outcomes resulting from different techniques (i.e., DTI, HARDI, and DSI from the same subjects) suggested: (1) that there is likely only a 15–20% difference between connectomes generated using the different acquisition and image reconstruction schemes, and (2) that while DTI acquisition and analysis techniques failed to reconstruct complex crossing fibers and therefore had lower sensitivity (i.e., higher Type II error), there were certain cases (e.g., short U-fibers) where DTI may even outperform the higher order HARDI and DSI models, which were more likely to have Type I errors owing to the inclusion of aberrant fibers (Rodrigues et al., 2013). However, although future tractography studies could reconstruct fibers with complex crossings and yield better sensitivity (i.e., lower Type II error)—e.g., by using higher *b*-values, more diffusion-encoding directions and more sophisticated reconstruction approaches than the single tensor model employed in our analyses—all current diffusion-based fiber tracking methods are inherently prone to both Type I (false positive) and Type II (false negative) errors. Given these limitations, emerging anatomical methods for mapping 3D networks—e.g., CLARITY (Chung et al., 2013)—may eventually be used to replace MRI-based atlases (including ours) altogether, but for now it remains to be seen whether advances in these techniques will overcome current barriers to studying intact white matter networks in whole human brains. Therefore, until arguably better diffusion imaging (e.g., HARDI, DSI, etc.) or 3D anatomical (e.g., CLARITY) white matter atlases supersede and replace them, the current atlases represent the first and best principled attempt to identify white matter regions associated with the functionally-defined Default Mode, Executive Control and Salience Networks.

However, one point that we feel cannot be overemphasized is that the limitations of the current atlases *must* be considered in any of their future applications (and the resulting interpretations and conclusions). Due to the fact that many real white matter connections were probably not identified in our tractography analyses (i.e., owing to Type II errors), the current atlases cannot be used to make claims about which regions are *not* part of a given tract or network. For example, based solely on our connectivity analysis between left BA22 and left BA44 (Supplementary Figure 1 and Supplementary Video 66), we cannot exclude the possibility that many voxels outside of our group probability map are also part of the left arcuate fasciculus (in fact, many other regions—particularly those in close proximity to the group probability map—likely are). However, using the same example, we suggest that the current atlases can be used to predict (with at least some measure of confidence) which white matter regions *are* part of the left arcuate fasciculus: and, by extension, the same goes for the DMN, ECN, and SN white matter group probability maps. Although there may be other appropriate applications within the confines of these limitations, we propose that the primary utility of these atlases will be for: (1) identifying whether white matter lesions *are* likely to be located within one or more of these networks, or (2) extracting quantitative white matter imaging metrics from various tracts/networks to allow the types of analyses shown in Figure 9.

It could be argued that one of the other limitations of the current study in particular, is that we only performed within-network tractography analyses for six functionally-connected brain networks (out of dozens of possible networks). These networks were chosen because the DMN, ECN, and SN are three of the most well-established and most studied intrinsically connected brain networks. Briefly, the DMN is comprised of a set of brain regions—including the medial prefrontal, medial temporal, and posterior cingulate cortices—that are both active and intrinsically connected with one another at rest (Gusnard and Raichle, 2001; Raichle et al., 2001; Raichle and Snyder, 2007) and anti-correlated with activity in several cortical regions involved in attentional control or cognitive processing (Fox et al., 2005; Fox and Raichle, 2007). The ECN, on the other hand, is comprised of nodes—throughout the prefrontal and parietal cortices, as well as the cerebellum—that are activated and synchronized during planning, inhibition, working memory, and other executive functions (Seeley et al., 2007; Bressler and Menon, 2010; Niendam et al., 2012). Finally, the SN—which is comprised of the dorsal anterior cingulate, orbitofrontal cortex, insula, and several other subcortical and limbic structures—is thought to play a significant role in emotional control (Seeley et al., 2007), cognitive control (Menon and Uddin, 2010), and error processing (Ham et al., 2013). Moreover, the SN is thought to be critically involved in switching between exogenous and endogenous attentional states and regulating the balance between DMN and ECN activity (Bressler and Menon, 2010). Therefore, in addition to being among the three most well-established intrinsically connected brain networks, the DMN, ECN, and SN appear to be inherently related to (and interconnected with) one another.

One additional limitation in the current study is that we did not examine any between-network connections (including the dDMN-to-vDMN, lECN-to-rECN, or aSN-to-pSN connections), and were therefore not able to generate probabilistic maps for these or other between-network ROI-to-ROI contingencies. While this would of course have been optimal (and may still happen in the future), the fact is that the number of ROI-to-ROI contingencies increases exponentially with the number of nodes, rendering it impractical to include the additional tractography analyses in the current study. For example, combining the dorsal and ventral DMN would result in 19 nodes (171 ROI-to-ROI contingencies), combining the left and right ECN would result in 12 nodes (66 ROI-to-ROI contingencies), and combining the anterior and posterior SN would result in 19 nodes (171 ROI-to-ROI contingencies), for a total of 408 ROI-to-ROI combinations. Across 32 subjects, this would require a staggering 13,056 tractography analyses (i.e., more than twice as many as the 6336 analyses performed in the current study). Perhaps this can be done in a future study using more automated analysis methods, but for now, this goes beyond the scope of the current manuscript.

## Conclusions

The landscape in systems and cognitive neuroscience has increasingly shifted from mapping the function of individual brain regions to investigating the functional connectivity within and between distributed, large-scale networks. Until now, however, there has been no principled method for measuring white matter changes and ascribing them to a specific network. By creating an extensive set of functionally-defined probabilistic white matter atlases (in stereotaxic coordinates), this study provides the first coherent framework for evaluating the microstructural integrity and white matter connectivity within the Default Mode, Executive Control and Salience Networks. Based on these atlases, future studies will be able to nominally attribute localized microstructural changes (either between groups or among individual patients) to a particular functional brain network, define specific tracts as *a priori* regions of interest within one or more of these networks, or investigate structure–function relationships that could provide deeper insights into the underpinnings of complex neural processes and/or disease.

### Conflict of interest statement

The Review Editor Pew-Thian Yap declares that, despite being affiliated with the same institution as the Associate Editor Charlotte A. Boettiger, the review process was handled objectively. The Review Editor Kenichi Oishi declares that, despite being affiliated with the same institution as the Author Susan Courtney, the review process was handled objectively. The authors declare that the research was conducted in the absence of any commercial or financial relationships that could be construed as a potential conflict of interest.

## Footnotes

^1^It is perhaps worth noting that previous tract-based white matter atlases have implemented FA > 0.15 and deviation angle thresholds above 40° for similar reasons (e.g., Wakana et al., 2004), while other deterministic tractography studies have used FA thresholds as low as 0.10 and deviation angle thresholds of 45° (e.g., Van den Heuvel and Sporns, 2011; Marqués-Iturria et al., 2015). Therefore, while the tractography parameters implemented in the current study are more liberal than the default values in DTIStudio, they are within previously established boundaries.

^2^These ROIs were originally identified by performing group independent component analysis (ICA) on rs-fMRI data from 15 healthy, right-handed control subjects between the ages of 18 and 30 years old (Shirer et al., 2012). They are shown in 2D in Figures 3–5 and Figures 7–9, in 3D in Supplementary Videos 1–6, and are included as Nifti images with the current white matter atlases (www.nitrc.org/projects/uofm_jhu_atlas).

^3^While performing conventional multi-ROI tractography in DTIStudio (i.e., using the more common “And” operation), the resulting streamlines may consist of three distinct regions. These include: (1) any regions where the streamlines project anterior to the anterior-most ROI; (2) the regions between the two ROIs; and (3) any regions where the streamlines project posterior to the posterior-most ROI. Alternatively, using the “Cut” operation only reconstructs the portion of these streamlines that lies between the two ROIs, cutting off the portions which extend in either direction beyond each ROI (for figures and a more detailed explanation of the “Cut” operation, please see Wakana et al., 2007).

^4^It is perhaps worth noting that the second type of voxel-wise “overlap analysis” can be used with conventional, non-quantitative imaging methods as well (e.g., FLAIR images) to estimate the specific white matter lesion load within each network, potentially providing clinical relevance for studying individual patients or patient populations (e.g., Multiple Sclerosis, Traumatic Brain Injury, etc.).

^5^Classical multidimensional scaling (also called principal coordinates analysis, Torgerson scaling or Torgerson–Gower scaling) is a well-established technique that can be used to: (1) reduce the dimensionality of a dataset, and (2) place each new data point into an N-dimensional matrix, while preserving the original distances between data points in the original dataset by minimizing a loss function (Borg and Groenen, 2005). Using this method, we were able to reduce the 3D coordinates of each cortical node's center of mass and represent them on a 2D figure (Supplementary Figure 2), while preserving the Euclidean distances between each node.

## Supplementary material

Supplementary Figure 1 **Binary masks of left BA22 (yellow), left BA44 (orange) and the resulting group probability map of the tractography streamlines identified across all subjects (purple)**. As expected, the topology of the identified streamlines was highly consistent with the left arcuate fasciculus, suggesting that our preprocessing pipeline and tractography parameters yielded streamlines with reasonable specificity (i.e., owing to the paucity of “spurious” streamlines). Moreover, the fact that streamlines were identified in 27 out of 32 (i.e., approximately 85% of) subjects—despite the fact that both of these BA masks were smaller and more restricted to cortical grey matter than our subsequent functionally-defined ROIs—suggests that the current approach also afforded good sensitivity. Also see Supplementary Video 66 for a 3D rendering of the group probability map in greater detail.

Supplementary Figure 2 **The connection counts (represented by line weight) for all of the ROI-to-ROI contingencies within each functionally-defined white matter network**. The line weights are the same as those shown in Figures 3–5. However, multidimensional scaling was used in this depiction to separate the ROIs within each network based on the Euclidean distances between their respective centers of mass. This shows that the white matter connection counts are not simply related to the distance between nodes (i.e., since some distal nodes have higher connection counts than certain proximal nodes, and vice versa). Combined with the large number of ROI-to-ROI contingencies showing high connection counts, this finding suggests that each of these intrinsically connected functional networks also has a highly organized set of underlying white matter structural connections.

Supplementary Figure 3 **Total white matter volume of each network, determined by combining the functionally-defined group probability maps for all tracts with a connection count greater than or equal to 8/32 (but without any additional thresholding of the probability maps themselves)**.

Supplementary Figure 4 **The amount of overlap between each functionally-defined white matter network (with the same masks used to calculate white matter volume in Supplementary Figure 3)**. The amount of overlap between each pair of white matter networks is expressed **(A)** as a raw volume (in mm^3^), or **(B)** relative to the size of each network on the x-axis (e.g., approximately 26% of the overall vDMN mask overlaps with the overall dDMN mask, etc.)

**All of the functionally-defined white matter atlases described in this article can be freely downloaded from the NITRC website (www.nitrc.org/projects/uofm_jhu_atlas)**.
