# Supplementary material for: Probabilistic atlases of default mode, executive control and salience network white matter tracts: an fMRI-guided diffusion tensor imaging and tractography study
Source: Front Hum Neurosci. 2015 Nov 3;9:585. doi: 10.3389/fnhum.2015.00585 (PMC4630538; doi:10.3389/fnhum.2015.00585)
Supplement: Supplementary Figure 1 — Binary masks of left BA22 (yellow), left BA44 (orange) and the resulting group probability map of the tractography streamlines identified across all subjects (purple). As expected, the topology of the identified streamlines was highly consistent with the left arcuate fasciculus, suggesting that our preprocessing pipeline and tractography parameters yielded streamlines with reasonable specificity (i.e., owing to the paucity of “spurious” streamlines). Moreover, the fact that streamlines were identified in 27 out of 32 (i.e., approximately 85% of) subjects—despite the fact that both of these BA masks were smaller and more restricted to cortical grey matter than our subsequent functionally-defined ROIs—suggests that the current approach also afforded good sensitivity. Also see Supplementary Video 66 for a 3D rendering of the group probability map in greater detail. [file All_Supplementary_Figures.PDF]

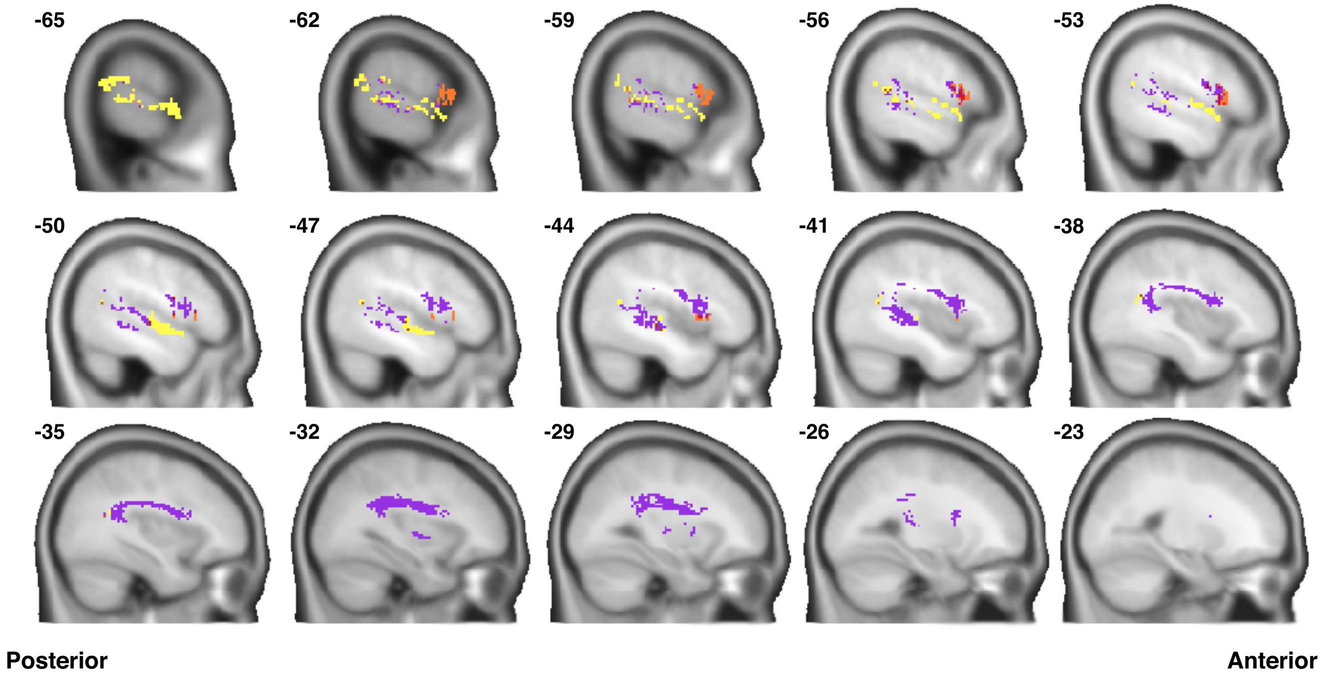

Supplementary Figure 1: Binary masks of left BA22 (yellow), left BA44 (orange) and the resulting group probability map of the tractography streamlines identified across all subjects (purple). As expected, the topology of the identified streamlines was highly consistent with the left arcuate fasciculus, suggesting that our preprocessing pipeline and tractography parameters yielded streamlines with reasonable specificity (i.e., owing to the paucity of 'spurious' streamlines). Moreover, the fact that streamlines were identified in 27 out of 32 (i.e., approximately 85% of) subjects – despite the fact that both of these BA masks were smaller and more restricted to cortical grey matter than our subsequent functionally-defined ROIs – suggests that the current approach also afforded good sensitivity. Also see Supplementary Video 66 for a 3D rendering of the group probability map in greater detail.

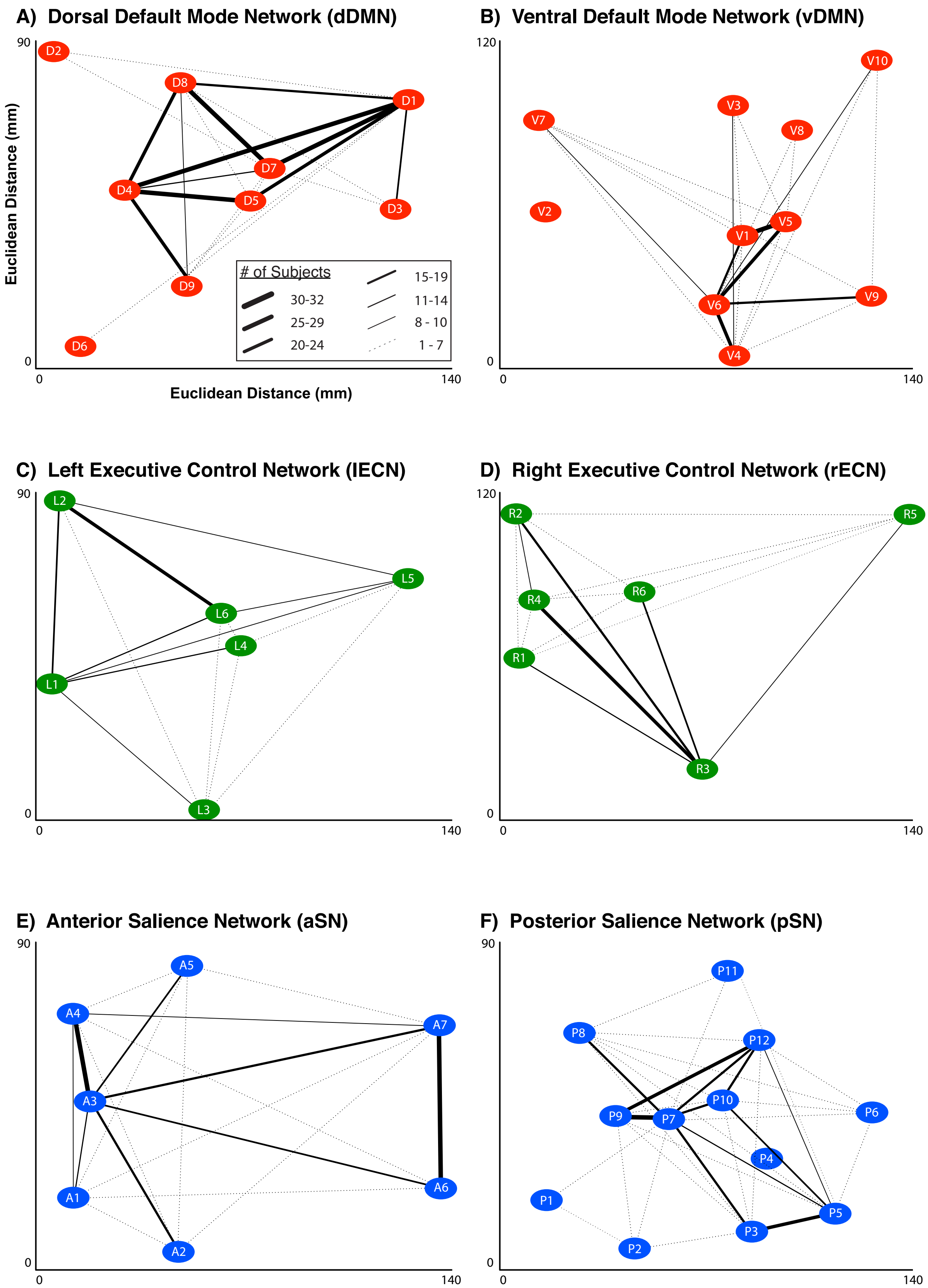

Supplementary Figure 2: The connection counts (represented by line weight) for all of the ROI-to-ROI contingencies within each functionally-defined white matter network. The line weights are the same as those shown in Figures 3-5. However, multidimensional scaling was used in this depiction to separate the ROIs within each network based on the Euclidean distances between their respective centers of mass. This shows that the white matter connection counts are not simply related to the distance between nodes (i.e., since some distal nodes have higher connection counts than certain proximal nodes, and vice versa). Combined with the large number of ROI-to-ROI contingencies showing high connection counts, this finding suggests that each of these intrinsically connected functional networks also has a highly organized set of underlying white matter structural connections.

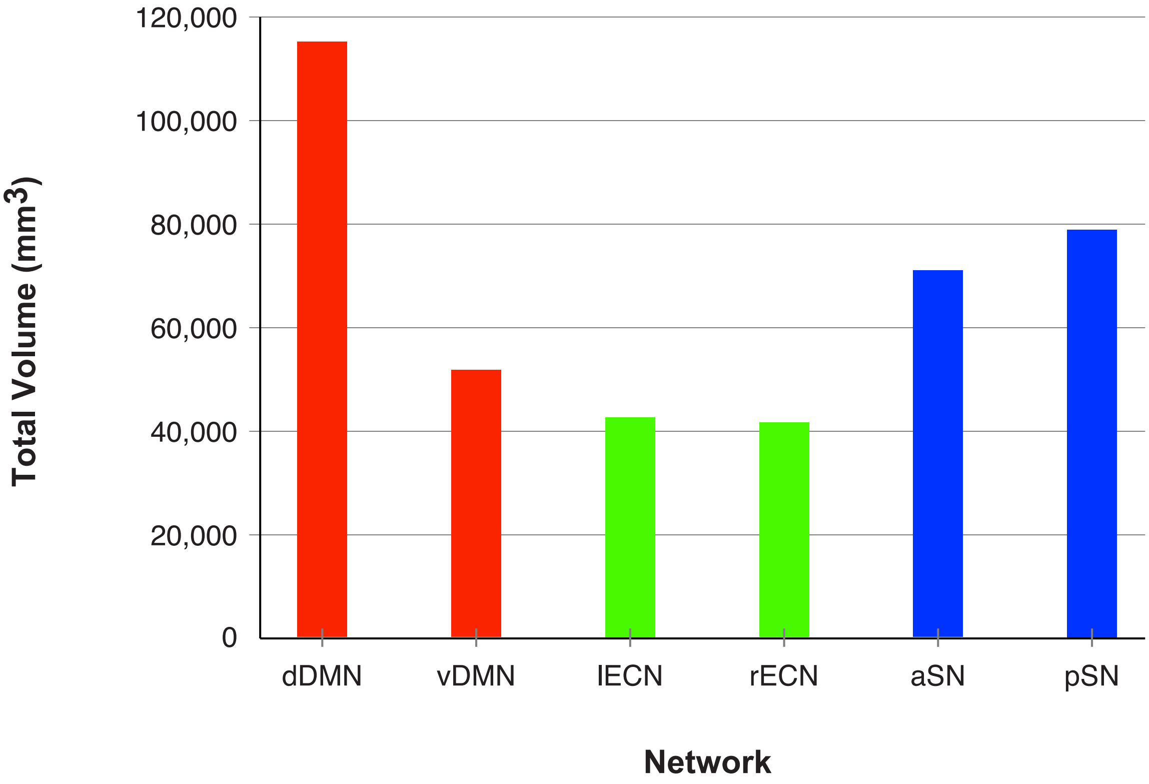

Supplementary Figure 3: Total white matter volume of each network, determined by combining the functionally-defined group probability maps for all tracts with a connection count greater than or equal to 8/32 (but without any additional thresholding of the probability maps themselves).

A) Overlap Between Networks (Raw Volume)

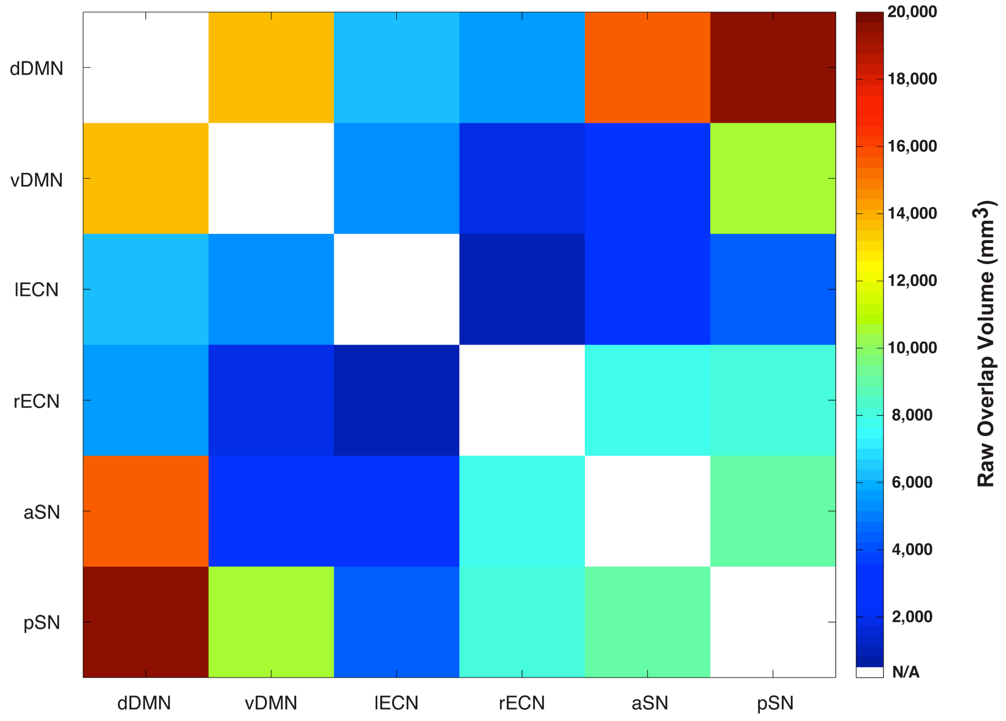

B) Overlap Between Networks (Normalized Volume)

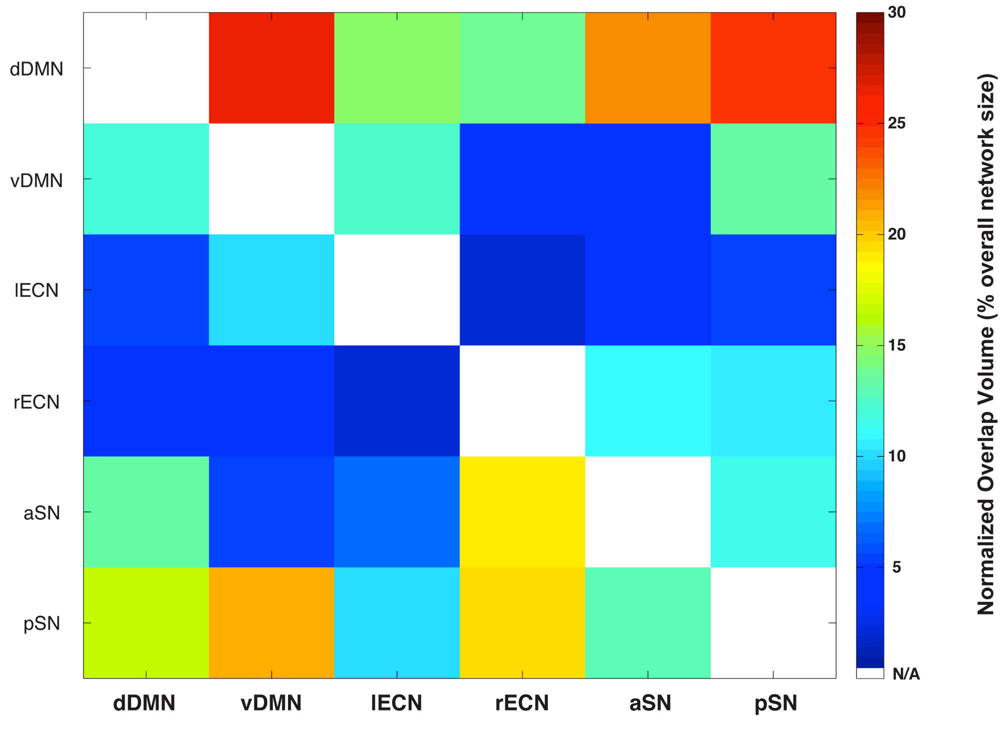

Supplementary Figure 4: The amount of overlap between each functionally-defined white matter network (with the same masks used to calculate white matter volume in Supplementary Figure 3). The amount of overlap between each pair of white matter networks is expressed **A)** as a raw volume (in mm^3), or **B)** relative to the size of each network on the x-axis (e.g., approximately 26% of the overall vDMN mask overlaps with the overall dDMN mask, etc.).
